# A cell suspension based uptake method to study high affinity glucosinolate transporters

**DOI:** 10.1186/s13007-020-00618-0

**Published:** 2020-05-24

**Authors:** Deepti M. Nambiar, Juhi Kumari, Gulab C. Arya, Amarjeet K. Singh, Naveen C. Bisht

**Affiliations:** 1 National Institute of Plant Genome Research, Aruna Asaf Ali Marg, New Delhi, 110067 India; 2 Department of Genetics, CGMCP, University of Delhi South Campus, New Delhi, 110021 India

**Keywords:** Cotton cell suspensions, Secondary metabolite transporters, Glucosinolates, GTR transporters, Kinetic analysis

## Abstract

**Background:**

Glucosinolates are an important class of secondary metabolites characteristic to the order Brassicales. They are known to play a major role in plant defense and from the human perspective, can be anticarcinogenic or antinutritive. GTRs are plasma-membrane localized high affinity glucosinolate transporters, which are important components of the source (leaf) to sink (seed) translocation of intact glucosinolates in members of Brassicaceae family. GTRs are identified as major candidates for *Brassica* crop improvement, thus dictating a need for their functional characterization. However, currently there are limitations in availability of heterologous assay systems for functional characterization of plant secondary metabolite transporters. To date, the animal-based *Xenopus* oocyte system is the best established heterologous system for functional characterization of these transporters. Inherent biochemical and physiological attributes unique to the plant membranes necessitate the need for developing plant-based transporters assay systems as well.

**Methods:**

In this study, *Agrobacterium* mediated transformation was used to develop *GTR* expressing cotton cell lines (CCL-1) for functional characterization of the *Arabidopsis* high affinity glucosinolate transporters, AtGTR1 and AtGTR2. Following sub-cellular localization of AtGTRs, we standardized the glucosinolate uptake assays using cell suspension cultures of *AtGTR* expressing CCL-1 its requirement of pH, salt, and time based glucosinolate uptake. Using the GTR expressing CCL-1, we subsequently performed kinetic analysis of AtGTR1 and AtGTR2 for different glucosinolate substrates, sinigrin, gluconapin and sinalbin.

**Results:**

Several clones expressing each of *AtGTR1* and *AtGTR2* were obtained showing high level of *GTR* expression and were maintained through regular sub-culturing. Both AtGTR1 and AtGTR2 are predominantly plasma-localized proteins when overexpressed in CCL-1 cells. Uptake assays were standardized, suggesting that glucosinolate uptake of GTR expressing CCL-1 is robust within the physiological pH range 5–6, and at lower concentration of nitrate salts. GTR expressing CCL-1 cells show increasing glucosinolate accumulation in time course experiment. Kinetic studies over a wide glucosinolate concentrations (10–800 µM) revealed that our novel assay system displayed robust GTR-mediated uptake of different glucosinolates and unambiguously helps elucidate the saturable kinetics of GTRs. Our system confirms the high affinity of AtGTRs for both aliphatic and aromatic glucosinolates.

**Conclusion:**

The transporter assay system described in this study holds potential for studying sub-functionalization amongst *GTR* homologs present across Brassicaceae family. The fast growing CCL-1 cells, confer the benefits of an in vitro system for quick assays and is plant based thus enabling optimal expression without sequence modifications. The efficient functioning of the GTR transporters in the heterologous CCL-1 opens the possibility of using this plant cell suspension system for functional characterization of other metabolite transporters.

## Background

A prerequisite for functional characterization of plant membrane transporters is a reliable and well standardized biological assay system. To date, yeast is an important heterologous system for plant transporter studies. Availability of mutant strains which lack functional homologues for certain plant primary metabolite transporters makes this system ideal for study of these transporters. The first plant transporter studied using this system was *Chlorella* HUP1 glucose transporter [20]. However, there are due marked differences between yeast and plant cells. Major problems which can be encountered while using this system for functional characterization of plant plasma membrane proteins are poor expression, retention in intracellular compartments leading to incorrect localization and altered translation which can cause aberrant functioning [4, 6]. There have been limited attempts towards using this system for study of plant secondary metabolite transporters such as the GTRs. A major hurdle was that detection of substrate uptake by the transporter under investigation required the utilization of the substrate or its metabolite for growth. In this context, attempts at genetic engineering of yeast with sulphatase gene from *Helix pomatia* or myrosinase gene from *Brassica napus,* in order to enable cells to use glucosinolate hydrolytic products as a Sulphur source for growth, have as yet been unsuccessful [24]. However, there have been recent reports of limited success towards functional characterization of Abscisic acid transporters using the yeast system. Detection of substrate uptake was enabled through coupling with mass spectrometry based techniques, thus providing scope for future utilization of the yeast system for study of plant secondary metabolite transporters [8].

The *Xenopus* oocyte is another robust system, currently also the best system for investigating plant secondary metabolite transporters. Glucosinolate transporters (GTRs) were first characterized using this system [14]. Oocytes being endowed with efficient protein translation machinery show optimum expression of the desired protein [13]. This system is amenable to electrophysiological experiments involving transporters since mature oocyte diameter ranges between the ideal values of 1.0–1.33 mm [3]. This system can be used to develop plant transporter cDNA expression libraries which help in identifying novel transporters [16]. However, fundamental differences such as a more negative resting membrane potential in plant cells and presence of endogenous voltage gated channels such as chloride channels in oocytes, render this system tricky in context of studying the plant membrane transporters.

A heterologous system which is poorly utilized, nonetheless holds promise for use for plant transporter study is the insect cell system. Recombinant Baculovirus infected Sf9 and Sf21 cell lines derived from *Spodoptera frugiperda* were used to investigate plant K + channels [5]. Insect cells show high expression of the foreign protein with the requisite post-translational modifications and are compatible with electrophysiological experiments. However, construction and purification of recombinant Baculovirus expression vectors are technically difficult. Moreover, poor documentation of endogenous channels and inability of insect cell membranes to withstand the highly negative membrane potential required for plant transporter study makes the system a less attractive option currently. In the last two decades, BY-2 plant cell system derived from tobacco has been used to investigate the role of plant transporters in biological processes such as auxin efflux and abscisic acid transporters [2, 9, 10], and holds scope for use in plant transporter characterization. Thus, no single system can suffice for functional characterization of plant transporters. Plant cell microenvironment is inherently different from animal cells and these differences can often translate into differences in expression, localization, regulation and function, particularly that of the membrane transporters. It is therefore desirable to have a plant based assay system for study of plant transporters such as the GTRs.

GTR1 and GTR2 are plasma membrane localized high affinity glucosinolate transporters first characterized in the model plant *Arabidopsis thaliana* [14]. Electrophysiological experiments characterized GTR transporters as H +/glucosinolate influx symporters. Based on sequence homology and mechanism of action, these transporters are categorized as members of the proton dependent oligopeptide (POT) family. Having been derived through neo-functionalization in nitrate transporters in course of evolution, they belong to the highly diversified NRT/PTR family. GTR have retained their affinity for nitrates and have high specificity for aromatic and aliphatic glucosinolates [7]. These transporters possibly transport phytohormones such as jasmonoyl-isoleucine and gibberellins [18]. GTR transporters play an important role in phloem loading, leaf distribution as well as rhizosecretion of glucosinolates [12, 14, 25]. These transporters have also been recognized as key players in glucosinolate accumulation in sulphur-rich S-cells in phloem cap cells [26]. They are thus important components of the glucosinolate source to sink translocation pathway. GTR transporters have been identified as potential targets in commercially important *Brassica* crops, for altering the glucosinolate profile towards improvement of the nutritive value of edible parts, without compromising on plant defence [15]. However, the presence of a vast repertoire of functional *GTR* homologs in polyploid *Brassica* crops, dictates the need for their functional characterization and structure–function analysis.

In this study, we describe a novel method for functional characterization of two well-known glucosinolate transporters, AtGTR1 and AtGTR2 by heterologously expressing them in a fast-growing cell-suspension line (CCL-1) derived from cotton, *Gossypium hirsutum*. The CCL-1 lacks endogenous glucosinolate biosynthetic machinery and thus GTR transporters. The *GTR* transformed cell lines show robust GTR mediated uptake of glucosinolates and are ideal for detailed kinetic studies. The plant cell suspension based uptake method described in this study holds promise for use in structure–function analysis of the diverse GTR transporters and possibly other metabolite transporters.

## Results

### Development of GTR expressing cotton cell lines

**Fig. 1:**
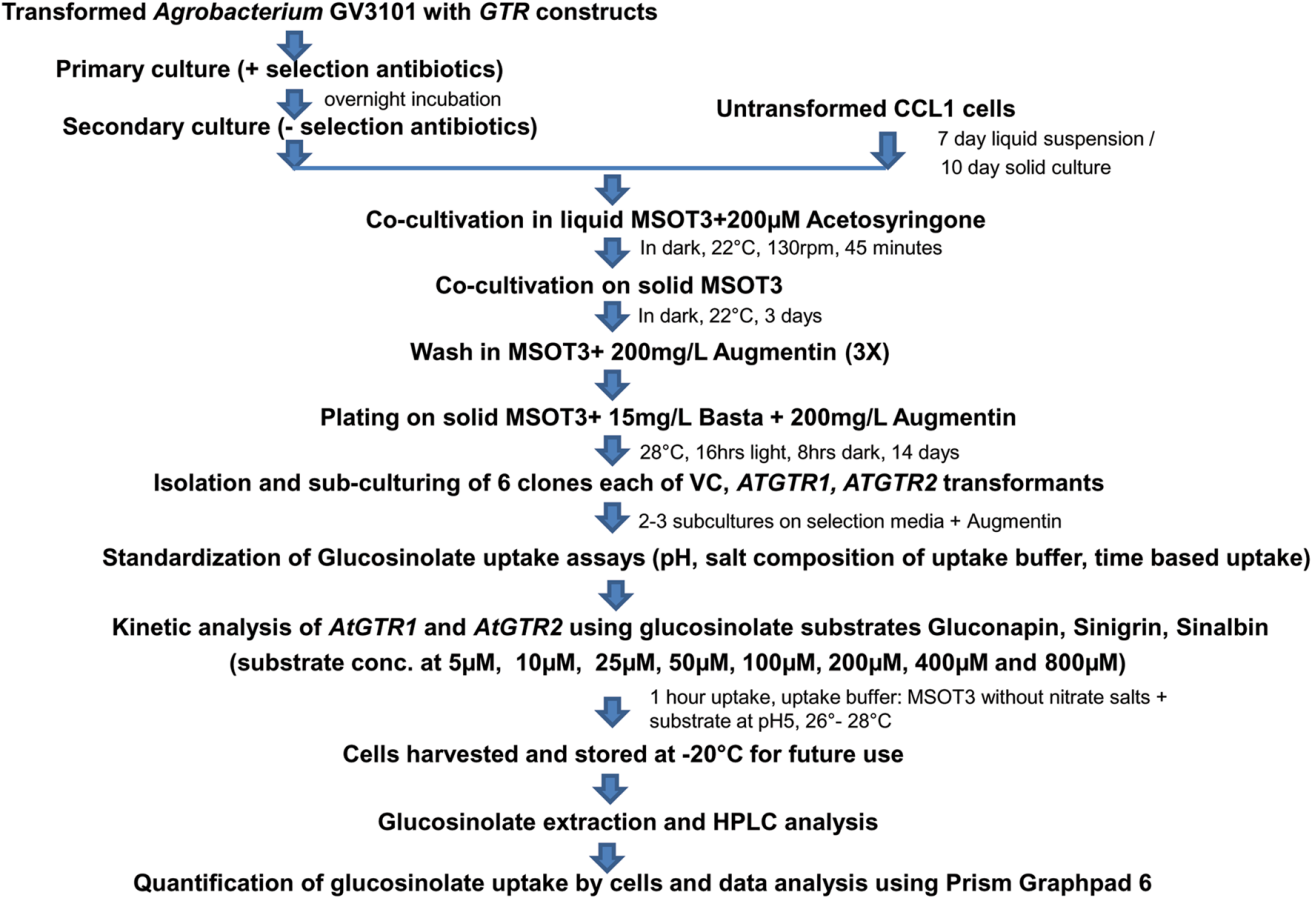
Flow chart depicting generation of GTR transformed cotton cell lines (CCL-1) and subsequent performance of glucosinolate uptake assays

**Fig. 2:**
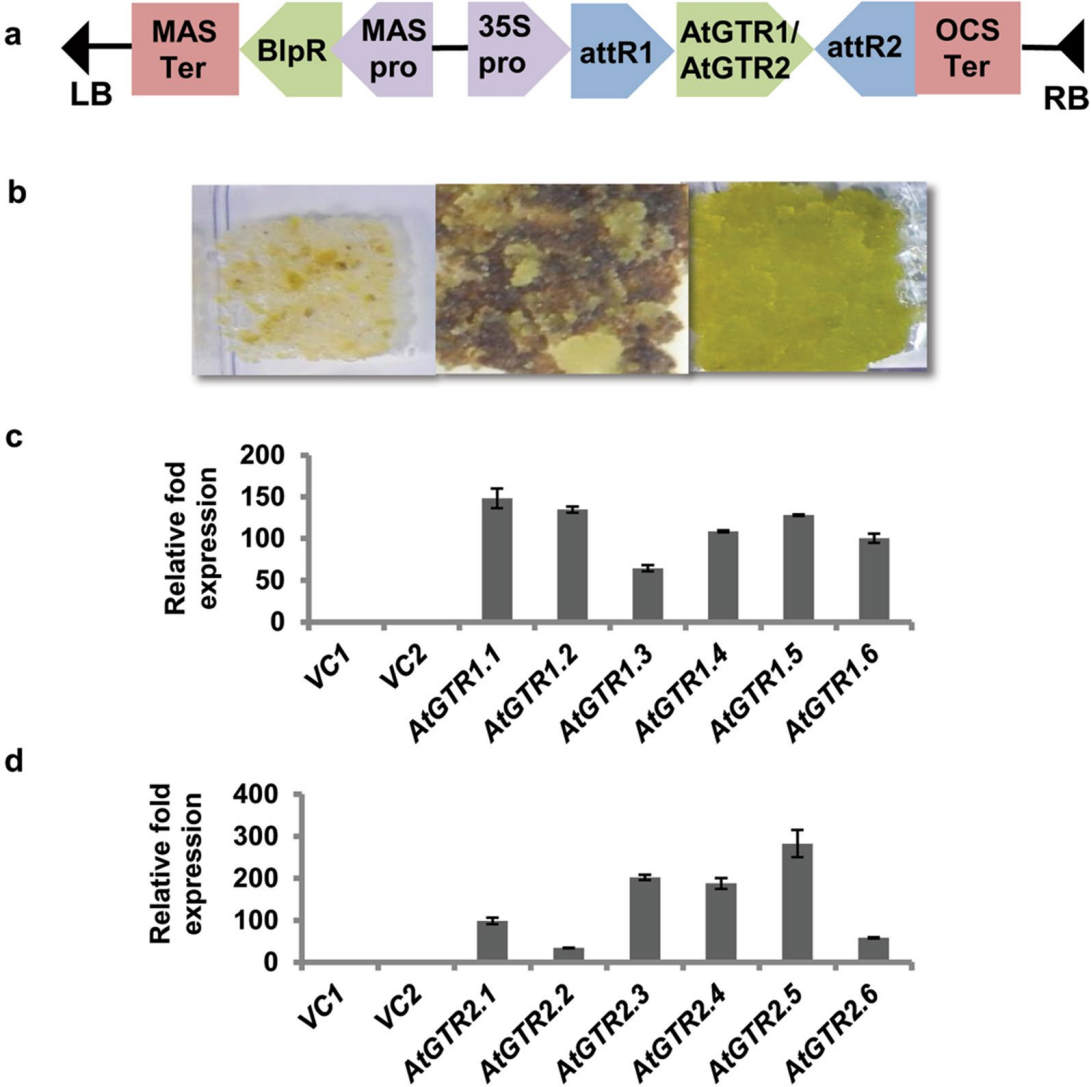
Generation and validation of GTR over-expressing CCL-1. **a** T-DNA map representing *GTR* over-expression construct. **b** Starting from left, untransformed cells on Basta selection media, *GTR* transformant clones growing through dead untransformed cells on selection media, *GTR* transformant clone maintained on selection media; (**c**) and (**d**) qRT-PCR analysis showing relative fold expression of *AtGTR1* and *AtGTR2* in CCL-1 transformants, respectively (w.r.t. cotton *Ubiquitin* set at 100). Six clones each of *AtGTR1* and *AtGTR2* over-expressing CCL-1 are presented along with vector controls (VC1 and VC2)

In order to functionally characterize the glucosinolate transporters (GTRs), we exploited the cotton cell-suspension line (CCL-1), which is a homogenous cell line derived from non-embryogenic calli of cotyledon origin [21]. A flow chart of the procedures adopted in the current study is depicted in Fig. 1. Briefly, *Agrobacterium* mediated transformation of the fast growing CCL-1 cells was performed with T-DNA constructs comprising of gene specific coding sequences (CDS) of Arabidopsis *AtGTR1* and *AtGTR2* under the CaMV35S constitutive promoter and Basta resistance gene (*BlpR*) as the selection marker (Fig. 2a). CCL-1 cells with T-DNA construct lacking any GTR insert served as vector control. Numerous putative transformants were obtained for each construct after two–three rounds of sub-culture on the selection media, at 14 days interval each (Fig. 2b). T-DNA insertion into genomes of putative CCL-1 transformants for vector control, *AtGTR1* and *AtGTR2* was first confirmed through amplification of *BlpR* gene from the genomic DNA of the mentioned, using gene specific internal primers (Table 2). Further, incorporation of the *AtGTR1* and *AtGTR2* genes was also checked through amplification of *AtGTR1* and *AtGTR2* using gene specific internal primers.

Relative expression levels of *AtGTR1* and *AtGTR2* genes were ascertained through qRT-PCR for all the transformants using gene specific internal primers, with the ubiquitin gene as the reference gene. The *AtGTR1* and *AtGTR2* transformant CCL-1 showed several fold higher relative expression of their respective transgenes, whereas no *GTR* expression was detected in the vector control transformants (Fig. 2c, d). Relative expression levels varied across clones, which could possibly be resulting from the transgene position effect. Following the confirmation of presence of the desired transgene and expression analysis of *GTRs* in CCL1 transformants, a total of six independent clones of each construct were selected and maintained.

**Fig. 3:**
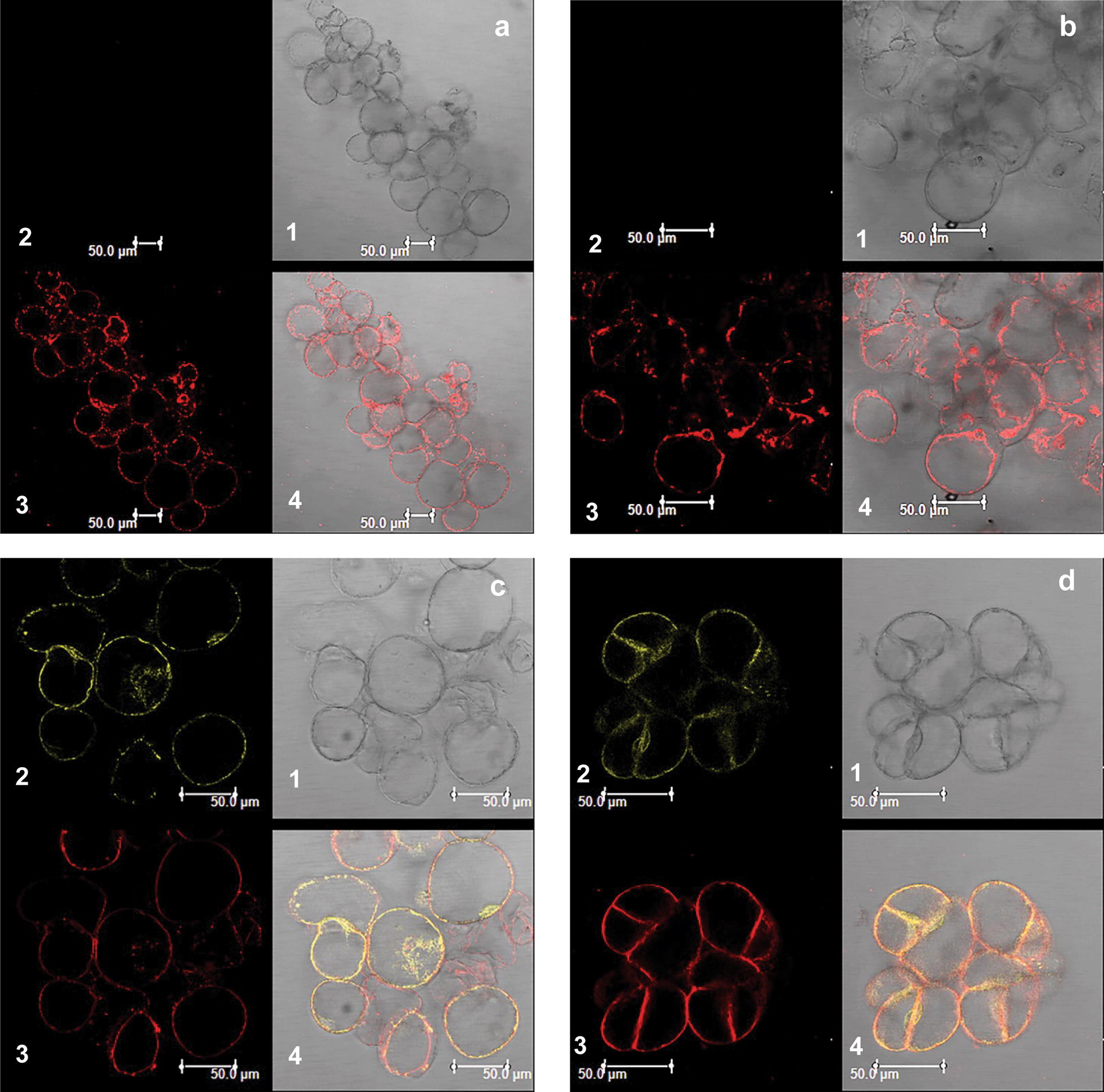
Sub-cellular localization of GTR transporters in CCL-1 cells. **a** Untransformed CCL-1 cells, **b** CCL-1 cells transformed with empty vector, pEarlygate101, **c** AtGTR1:YFP expressing CCL-1 cells, and (**d**) AtGTR2:YFP expressing CCL-1 cells. For each of these panels, the bright field (1), YFP fluorescence (2), FM4-64 marker fluorescence (3), and merged (4) images are arranged in the anticlockwise order from top right

The TMHMM server 2.0 software (http://www.cbs.dtu.dk/services/TMHMM/) predicts AtGTR1 and AtGTR2 as 12 transmembrane (12-TM) proteins. To prove the usefulness of CCL-1 as a functional analysis of transporters, we investigate the subcellular localization of AtGTR1 and AtGTR2 proteins in the heterologous system. The Pro35S:GTR1:YFP and Pro35S:GTR2:YFP fusion constructs were stably transformed in CCL-1 cells using the *Agrobacterium* transformation protocol. Untransformed CCL-1 cells and empty vector control (lacking functional YFP) served as negative controls and showed no YFP fluorescence. The GTR transformed CCL-1 cells showed a strong yellow fluorescence signal which co-localizes with the staining of the FM4-64 membrane marker dye (Fig. 3), thus demonstrating that both AtGTR1 and AtGTR2 are predominantly plasma-localized proteins, when overexpressed in CCL-1 cells.

### Effect of nitrate salts in uptake buffer

Functional analysis of the *GTR* cell lines required prior standardization of the uptake buffer. The MSOT3 growth medium used for maintaining CCL-1 cultures comprises of a carbon source as well as an osmoticum, both being the minimal requirements for maintaining the integrity and health of the cell suspensions during the uptake assays. However, the growth medium also contains nitrate salts in abundance (30 mM KNO_3_). Since GTR transporters have been proven to retain their affinity for nitrates, it was important to check the effect of nitrates on the transport efficiency (expressed as nmoles/g/hour) through checking glucosinolate uptake by the *AtGTR* transformed cells when the growth medium was used as an uptake buffer.

**Fig. 4:**
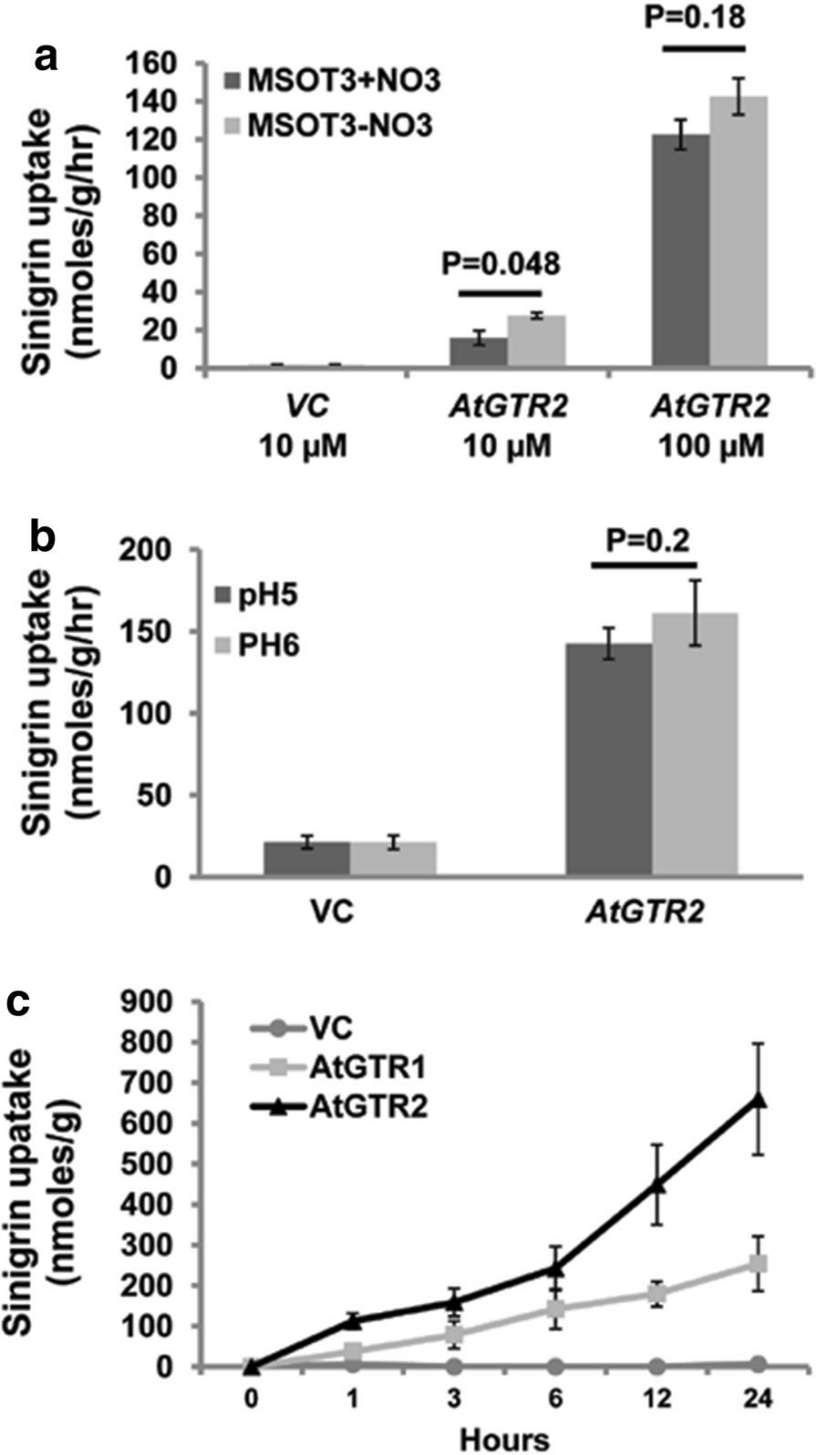
Standardization of transport uptake assays using AtGTR expressing CCL-1. **a** Effect of presence of nitrate salts in millimolar range in uptake buffer on uptake efficiency using ATGTR2 expressing CCL-1. The uptake was performed at 10 µM and 100 µM of sinigrin in uptake buffer for 1 h. **b** Effect of pH of uptake buffer on uptake efficiency using AtGTR2 expressing cells. **c** Time based uptake of sinigrin through 0–24 h duration. Uptake assays for three independent clones were performed with sinigrin at 100 µM for 1 h, unless indicated. Data represents mean ± SE (n = 3), and student’s t-test, two tail distribution was performed with a threshold p value of 0.05

Uptake assays were performed on vector control and *AtGTR2* expressing cells using MSOT3 growth media directly as the uptake buffer with 10 µM and 100 µM sinigrin, an aliphatic glucosinolate. Alternately these uptake assays were performed using MSOT3 without nitrate salts as the uptake buffer. No detrimental effect of nitrate salts elimination was observed on the integrity of the CCL-1 plasma membrane. Notably, the glucosinolate uptake by *AtGTR2* expressing cells was reduced markedly to almost 50% when MSOT3 was used as the uptake buffer at a lower concentration of sinigrin (10 µM) as compared to MSOT3 without nitrates (Fig. 4a). However, no significant reduction was recorded in assays performed at 100 µM sinigrin.

### Effect of pH of uptake buffer on assay efficiency

Another relevant parameter pertaining to the uptake efficiency of the cells was the pH of the uptake buffer. GTR transporters are dependent on protons for symporting glucosinolates. It was reported that for maximal glucosinolate uptake by *Xenopus* oocytes, the uptake buffer was required to be at pH5 [14]. Thus, to check if this held true for our system, uptake assays were performed using *AtGTR2* expressing CCL-1 cells, with uptake buffers at pH5 and pH6 and 100 µM sinigrin as the substrate. It was observed consistently that the cells stayed healthy and showed optimum growth at both pH5 and pH6 ruling out the possibility of erroneous results due to stress induced physiological alterations in the cell membrane. Our results showed no significant difference in GTR mediated sinigrin uptake efficiency at pH5 and pH6 (Fig. 4b).

### GTR expressing CCL-1 cells show time based glucosinolate uptake

Time based uptake assays using 100 µM Sinigrin shows that the glucosinolate is accumulated by cells sufficiently thus enabling detection from 30 min onwards. The uptake of glucosinolates by *AtGTR1* and *AtGTR2* expressing cells at a sinigrin concentration of 100 µM in the transport buffer was determined at regular intervals for the duration of 24 h. The ‘0’ hr time point represents the ‘control’ time point at which sinigrin was not added, no sinigrin peaks were detected clearly proving the absence of any endogenous sinigrin. Uptake and the consequential time dependent accumulation of sinigrin in *AtGTR1* and *AtGTR2* expressing cells are represented by a linear graph during the time range of 0–24 h (Fig. 4c). *AtGTR1* expressing cells showed an average sinigrin content of 37.9 ± 10.6 nmoles/g after 1 hour uptake, and its accumulation in the cells reaching 253.97 ± 67.3 nmoles/g after 24 h. Similarly, *AtGTR2* expressing cells showed an average sinigrin accumulation of 112.02 ± 19.8 nmoles/g after 1 hour uptake, the level rising to 659 ± 137.4 nmoles/g after 24 h.

### Functional and biochemical analysis of *Arabidopsis* GTRs using CCL-1

**Fig. 5:**
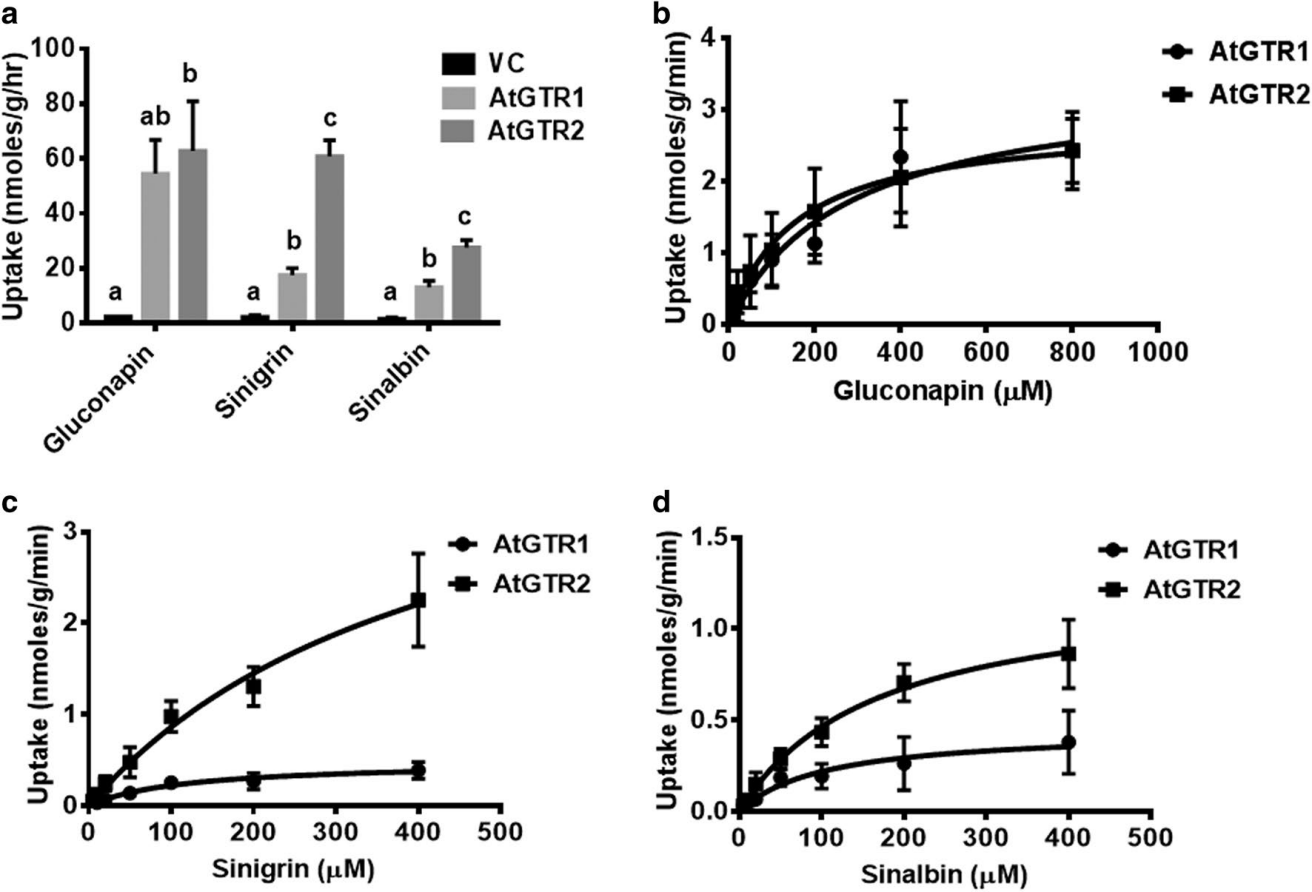
Functional analysis of AtGTR expressing CCL-1 cells **a** Hourly uptake of sinigrin at 100 µM conc. by vector control, AtGTR1 and AtGTR2 expressing CCL-1 cells. Tukey posthoc test in One-way ANOVA was performed to check for statistically significant difference in uptake between the different lines (n ≥ 3). **b** Kinetic analysis of AtGTR transporters for gluconapin **c** sinigrin, and **d** sinalbin. Uptake assays for three independent clones (100 mg) were performed with glucosinolate concentration (5–800 µM) for 1 h. Data represents mean ± SE (n ≥ 3)

Standardization of the uptake conditions was followed by functional analysis of the *GTR* expressing CCL-1. Uptake assays were initially performed using three different glucosinolate substrates, namely sinigrin, gluconapin and sinalbin at 100 µM glucosinolate concentration. *AtGTR1* and *AtGTR2* expressing cells showed several folds higher uptake of glucosinolates as compared to vector control, which showed low basal transport (Fig. 5a). Assays using 100 µM gluconapin as substrate showed *AtGTR1* and *AtGTR2* expressing cells accumulating gluconapin at an average of 54.5 ± 12.4 nmoles/g/hr and 62.9 ± 18 nmoles/g/hr respectively as compared to 2.4 nmols/g/hr for the vector control. 100 µM sinigrin showed an average sinigrin accumulation of 17.6 ± 2.5 nmoles/g/hr and 60.9 ± 5.8 nmoles/g/hr by *AtGTR1* and *AtGTR2* expressing cells respectively compared to 2.3 ± 0.6 nmoles/g/hr for the vector control. Further, in uptake assays with 100 µM sinalbin *AtGTR1* and *AtGTR2* expressing cells showed an average sinalbin accumulation of around 13.2 ± 2.3 nmoles/g/hr and 27.7 ± 2.6 nmoles/g/hr respectively as compared to 1.6 ± 0.5 nmols/g/hr of vector control. The *AtGTR* expressing cells showed robust uptake of both aliphatic (sinigrin, gluconapin) and aromatic (sinalbin) glucosinolates, confirming the ability of these transporters to transport both types of glucosinolates.

Since *GTR* transporters have a wide substrate range, kinetic analysis of these transporters can elucidate on any preferential substrate specificities. Thus, we performed kinetic analysis for AtGTR1 and AtGTR2 for glucosinolate substrates sinigrin, gluconapin and sinalbin (Fig. 5). Kinetic assays were performed over a concentration range of 10-800 µM for gluconapin and a range of 10–400 µM for sinigrin and sinalbin. Three to four clones representing independent transformation events and showing comparable transport efficiencies of each of the three glucosinolate substrates were selected for each of *AtGTR1* and *AtGTR2* expressing cell lines for kinetic analysis. The glucosinolate uptake by GTR transporters showed saturable kinetics and fitted into the Michaelis–Menten curve (Fig. 5b–d), as has already been reported using oocyte assay [14]. Km values and Vmax values for AtGTR1 and AtGTR2 transporters for all of the above mentioned glucosinolate substrates were obtained using our system (Table 1). Km values for AtGTR1 for sinigrin, gluconapin and sinalbin ranged between 104.5 µM and 279.1 µM. Further, maximum rate of either glucosinolate substrate uptake by cell lines expressing *AtGTR1* ranged between 0.5 nmoles/g/min and 3.4 nmoles/g/min. Km values for AtGTR2 for the three substrates ranged between 166.7 µM and 436.7 µM. The maximum rate of either glucosinolate substrate uptake by cell lines expressing *AtGTR2* ranged between 1.2 nmoles/g/min and 4.6 nmoles/g/min. The Km values for glucosinolates were in the micromolar range which is expected for high affinity transporters as is the case with these transporters. Moreover, both AtGTR1 and AtGTR2 transporters appeared to have high affinity for both aliphatic and aromatic glucosinolates. Taken together, our study provides a novel strategy for performing a detailed kinetic analysis of transmembrane proteins under in vivo condition using a plant derived CCL-1 cell line.

**Table 1:** Biochemical characterization of Arabidopsis GTRs for different glucosinolate substrates using the cotton cell line system. Uptake assays for three independent clones (100 mg) were performed with glucosinolate concentration (5–800 µM) for 1 h. Data represents mean ± SE

Substrate	Transporter	Km (µM)	Vmax (nanomoles/g/min)
Gluconapin	AtGTR1	279.1 ± 109	3.4 ± 0.6
Gluconapin	AtGTR2	153.3 ± 59	2.9 ± 0.4
Sinigrin	AtGTR1	117.9 ± 44	0.5 ± 0.07
Sinigrin	AtGTR2	436.7 ± 154	4.6 ± 1.1
Sinalbin	AtGTR1	104.5 ± 56	0.5 ± 0.09
Sinalbin	AtGTR2	166.7 ± 40	1.2 ± 0.1

## Discussion

We generated *GTR* expressing CCL-1 lines (*AtGTR1* and *AtGTR2* expressing cells) and standardized glucosinolate uptake assays for subsequent kinetic experiments (Figs. 1 and 2). Our experiments indicated that the MSOT3 culture medium without nitrate salts was an ideal uptake buffer. As already mentioned GTR transporters are phylogenetically related to nitrate transporters and have retained the ability to transport nitrates when present in millimolar ranges [14]. The presence of very high concentration of nitrates (30 mM) in the MSOT3 growth medium can compete with glucosinolates uptake at low glucosinolate concentrations (Fig. 4a). However, since GTR transporters are high affinity glucosinolate transporters the competitive effect of nitrates is not significant at a higher concentration of 100 µM. This aspect becomes relevant while performing kinetic assays in which depressed uptake at lower concentrations owing to competition from nitrates can yield exaggerated Km values.

Another important parameter on which the uptake efficiency might depend on was the pH of uptake buffer. GTR transporters are proton dependent and *Xenopus* assays for the GTRs indicated a distinctly higher uptake of glucosinolates at uptake buffer at pH5 compared to that at pH6 [14]. For our system, glucosinolate uptake efficiency was similar at uptake buffer pH5 and pH6 (Fig. 4b). This inconsistency might find a possible explanation in some key inherent differences between plant and animal cells. Plant cells have cell walls which play a key role in stress responses and growth thus requiring homeostatic regulation of the cell wall pH and ionic composition [19]. Moreover the plasma membrane ATPase ionic pumps in plants are H + -ATPases whereas animal cells predominantly use Na + , K + -ATPases. Extrusion of protons by plasma membrane H + -ATPases and homeostatic regulation cause acidification of the cell wall, creating an in built proton gradient across the membrane leading to a highly negative resting membrane potential of up to (-)200 mV [17]. This characteristic is especially true for plant cells involved in nutrient uptake [22]. In nature this feature helps energize proton dependent secondary active transporters such as nitrate, peptide and glucosinolate transporters (GTRs), the activity of which ensure sustenance of optimal growth and development of the plant. CCL-1 cultures which have a high nitrate requirement derive their nutrients directly from the growth media in the absence of a specialized vascular system. They also exhibit a similar rate of growth at both pH5 and pH6. They are thus expected to homeostatically maintain a natural transmembrane proton gradient within the narrow pH range of 5–6 of the growth media. On the other hand, use of animal cells such as *Xenopus* oocytes or even plant protoplasts for such assays, would require the creation of a transmembrane proton gradient through maintenance of a specific low pH of the uptake buffer.

A major advantage of the CCL-1 system is the ease of transformation, maintenance and use for experimentation. Moreover, since these are individual cells, they confer the advantage of an in vitro system. In context of biochemical characterization of GTR transporters, this system enables the study of the transport mechanism of GTR transporters in isolation of any interacting/influencing biological pathways. The CCL-1 cells are fast growing and easy to manipulate. These cells stay healthy in liquid suspension cultures for up to 7 days thus permitting considerable flexibility to the system in context of duration and design of transport assays. Cultures on solid media grow fast and stay healthy for up to 15 days from sub-culturing, thus being available for experiments for a considerable duration.

Since the CCL-1 cells are plant based, optimum heterologous expression towards ensuring robust functioning of *GTRs* required no prior modifications of the *GTR* CDS such as codon optimization and use of specific UTRs. Further, confocal imaging of both C-terminal YFP tagged GTR expressing CCL-1 cells respectively, confirmed plasma membrane localization of the GTRs. Functionally, *AtGTR* expressing cells showed robust GTR mediated glucosinolate uptake. Cells harvested prior to glucosinolate addition at 0 h time points showed an absolute lack of endogenous glucosinolates. Time based glucosinolate uptake assays clearly indicated a linear accumulation of sinigrin with increasing time, suggesting an unambiguous role played by a carrier in the accumulation of sinigrin. Since the accumulation of sinigrin is several folds higher in case of *AtGTR1* and *AtGTR2* expressing cells as compared to the vector control at every time point, it can be safely concluded that the accumulation of glucosinolates in the CCL-1 transformed cell lines is the result of AtGTR1 and AtGTR2 mediated transport. Time based assays also proved that long term exposure to glucosinolate had no toxic effect on the CCL-1 cells.

Further, kinetic experiments using the *GTR* overexpressing CCL-1 lines also reveal the suitability of the system for functional characterization of these transporters. Functional efficiency varied between different clones possibly arising from the transgene position effect. High functioning clones exhibited high GTR mediated glucosinolate uptake consistently through several rounds of subculturing. A single transformation process of CCL-1 cells can yield numerous clones representing independent transformation events. Since transport assays are easy to perform and require a small amount of tissue, high functioning clones can be selected and maintained through regular sub culturing for reproducibility through all subsequent assays. In case of *Xenopus* oocytes, heterologous expression of a protein varies across oocytes of different stages of development as well as different batches [13]. Besides, clamping the plasma membrane at highly negative voltage of (−)200 mV in case of electrophysiological experiments involving electrogenic plant transporters can activate endogenous voltage gated channels such as chloride channels [13] resulting in noise in the data. Thus reproducibility of results using this system depends on sound technical expertise towards selection of the right maturation stage of the oocytes, choice of appropriate controls, standardization of experiments and normalization of data. It should however also be noted that 1-hour uptake duration for kinetic analysis is expected to add a linear component to the data, as a result of time based accumulation of substrate, thus leading to slightly exaggerated Km values. The CCL-1 system is highly compatible with the sensitive liquid chromatography based detection systems (e.g. LC–MS) thus enabling accurate quantification of the substrate transport during early time points with limiting cell amounts, for a precise kinetic analysis.

The major findings using the system broadly conform to those obtained through investigation with established transport assay systems. *Xenopus* oocytes are large animal cells, a major fraction comprising of the cytosol whereas CCL-1 cells are typical plant cells characterized by a large vacuole and thin cytoplasmic lining surrounding the nucleus. Plasma membrane localized GTR transporters transport glucosinolate substrates into the cytosol [14]. Therefore, it is not possible to obtain a sharp comparison for robustness of the GTR mediated glucosinolate uptake between the two systems. Overall, the biochemical characteristics of AtGTR1 and AtGTR2 transporters elucidated through transport assays performed using our CCL-1 system are consistent with those obtained through functional characterization of these transporters using the *Xenopus* system [7, 14]. The transporters exhibit saturable kinetics, fitting perfectly into the Michaelis–Menten curve. These transporters showed high affinity for both aliphatic and aromatic glucosinolates with Km values for both substrates in µM ranges. The ability of nitrates to competitively reduce glucosinolate uptake by GTR transporters is also consistent with observations in the previous study. However, key inherent differences between heterologous systems stemming from their different biological origins make it difficult to draw detailed comparisons [13]. This is because major biochemical differences, such as that in the resting membrane potential, are expected in the plasma membranes of the plant based CCL-1 cells and animal based *Xenopus* oocytes. The differences between the plant based and animal based systems are also reflected in the pH dependency of the *Xenopus* and CCL-1 transport assay systems. The CCL-1 cells appear to offer the microenvironment closest to that in which GTRs function in nature. However, a limitation to our system is that it is not suitable for determining the proton dependence of plant transporters.

Due to efficient functioning of the AtGTRs, the CCL-1 system would be a suitable heterologous system for functional characterization of transporters encoded by different *GTR* homologs. This plant cell system is thus well suited for elucidating possible sub-functionalization between *GTR* paralogs and orthologs present in *Brassica* species. Moreover this system shows scope of use for establishing structure function relationships through mutational studies. Plant transporters are very diverse and their correct expression and functioning can be governed by numerous underlying biological aspects, thus creating a requirement for different heterologous expression systems to choose from. Due to all the mentioned advantages of the CCL-1 system, this cotton cell line system might hold promise for use in functional characterization of other plant secondary metabolite transporters such as phytohormone transporters. No phytohormones are required in the CCL-1 culture media. Being a plant based system, the CCL-1 cells might have some basal level of endogenous plant hormones which can be ascertained through vector controls towards subsequent data normalization. This was the strategy used for successful functional characterization of abscisic acid and auxin transporters in [10] and [2] respectively using the plant based BY-2 system.

## Conclusion

We have developed a novel plant based heterologous system for functional characterization of GTR transporters. We developed GTR overexpressing cotton cell lines, which showed robust GTR mediated glucosinolate uptake and can be maintained on solid media or as liquid cell suspensions. Due to their high GTR mediated glucosinolate uptake efficiency, they are ideal for kinetic studies and elucidation of differential substrate specificities of GTR homologs. Thus, the CCL-1 system is well suited for studying possible sub-functionalization between *GTR* paralogs and orthologs in polyploid *Brassica* species. Moreover, the CCL-1 system holds promise for use in investigation of membrane transporters for secondary metabolites.

## Methods

### Isolation and cloning of *Arabidopsis GTR* transporters

Total RNA was isolated from 3 weeks old Arabidopsis Col-0 leaves using Spectrum Plant Total RNA Kit (Sigma). cDNA was synthesized from the extracted RNA using Primescript First Strand cDNA Kit (Takara). Coding sequences (CDS) of *AtGTR1* and *AtGTR2* genes were amplified using gene specific primers (Table 2) from the cDNA with Phusion High Fidelity DNA polymerase (ThermoFisher Scientific), cloned into pENTR/D-TOPO directional cloning entry vector and confirmed using sequencing. The *AtGTR1* and *AtGTR2 CDS* were subsequently mobilized into pEarleyGate100 vector through gateway cloning. The destination vectors containing the *AtGTR1* and *AtGTR2* inserts were finally transformed into *Agrobacterium tumefaciens* GV3101. The pEarleyGate100 vector was used as the control construct.

**Table 2:** List of primers used in this study

S. no.	Primer	Sequence (5′-3′)
Gene amplification		
1	At3G47960 GTR1 FP	CACCATGAAGAGCAGAGTCATTCTTAACC
2	At3G47960 GTR1 RP	TCAGACAGAGTTCTTGTCTTGTAGC
3	At5G62680 GTR2 FP	CACCATGGAGAGAAAGCCTCTTGAAC
4	At5G62680 GTR2 RP	TCAGGCAACGTTCTTGTCTTGCTG
Transgene insertion validation		
5	Bar FP	GCTCTACACCCACCTGCTGAA-3′
6	Bar RP	TCAGATCTCGGTGACGG-3′
7	GTR1 internal FP	ATCAACAGTTTCTTCAACTGGT
8	GTR2 internal FP	TGGGGCTGATCAGTTTAACC
9	GTR1/GTR2 internal RP	ACTGCTTGTAGTAAAACTCCAT
Transgene expression validation		
10	AtGTR1(RT) FP	GAATCGGAGCTGGGTTTACA
11	AtGTR2(RT) FP	TGGCATTTTCTTCGCTACCGC
12	GTR1/GTR2 (RT) RP	ACTGCTTGTAGTAAAACTCCAT
13	Ubuiquitin FP	GGTGGGATGCAAATCTTCGTGAAAAC
14	Ubuiquitin RP	CTGGATGTTGTAGTCGGCCAAGGTA

### Maintenance and transformation of CCL1

The fast growing cotton cell lines (CCL-1) were obtained from Dept. of Genetics, Univ. of Delhi, South Campus. These cell lines are derived from nonembryogenic calli originating from cotyledons of *Gossypium hirsutum* var Coker310FR [21]. They are maintained as friable calli on solid MSOT3 media, pH5.8 at 28 °C, 16 h light and 8 h dark with no phytohormone requirement. Solid MSOT3 media is MS media with additional KNO3 (1.9gm/L) and 0.2% Phytagel. Alternately, 500 mg of CCL-1 calli can be inoculated in 50 ml liquid MSOT3 in 250 ml flask and maintained at the afore-mentioned conditions on an orbital shaker at 130 rpm.

Cell lines were transformed as mentioned in [21]. Briefly, 7 day old liquid cell suspensions or 10 day old solid cotton cell cultures are ideal for transformation. Approx. 500 mg of cotton cells was co-cultivated with *Agrobacterium* in liquid MSOT3 containing 200 µM Acetosyringone in dark at 22 °C for an hour followed by co-cultivation on solid MSOT3, pH5.8 for 3 days in dark at 22 °C. Finally cells were washed three times with liquid MSOT3 media containing 200 mg/L Augmentin; plated on solid media containing Basta (15 mg/L) and Augmentin (250 mg/L); and kept at 28 °C, 16 h light and 8 h dark. After 2 weeks transformants were visible as yellow and green calli.

These calli were sub-cultured every fortnight. Transformants were confirmed by isolating genomic DNA using CTAB method and PCR amplification using *BplR* gene, *AtGTR1* and *AtGTR2* specific internal primers (Table 2). Gene expression was ascertained through qRT-PCR. RNA was isolated from 200 mg of transformed cells using Trizol method, cDNA was synthesized using High Capacity cDNA Reverse Transcription Kit (ThermoFisher Scientific). *AtGTR1* and *AtGTR2* specific internal primers were used for qRT-PCR and relative expression was quantified using cotton *Ubiquitin* as the reference gene.

### Subcellular localization of AtGTR1 and GTR2 proteins

To generate a proCaMV35S:AtGTR1/2:YFP fusion construct, AtGTR1 and AtGTR2 CDS lacking the stop codons were mobilized to the C-terminal YFP fusion vector, pEarlygate101. Constructs were transformed into CCL-1 cells through *Agrobacterium* mediated transformation. CCL-1 cells expressing tagged proteins were teased in MSOT3 medium on a slide and stained with FM4-64 membrane marker dye at a working concentration of 2 µg/ml (Life Technologies, ThermoFisher). The slides were imaged immediately after staining, with the Leica TCS SP5 confocal microscope using appropriate filters for YFP (514/527 nm) and FM4-64 (515/640 nm). CCL-1 cells containing empty vector pEarlygate101 (with non-functional YFP) was used as the negative control.

### Glucosinolate uptake assays by *GTR* transformed CCL1

For glucosinolate uptake assays both 7–15 days old healthy callus cultures and 7 day old liquid suspension cultures can be used. Liquid MSOT3 without nitrate salts at pH5 was used as transport uptake buffer. For time based uptake assays, cell suspensions of transformed cell lines was obtained by adding 6 g cells in 30 ml MSOT3, pH5 in 250 ml conical flasks and kept on an orbital shaker at 130 rpm, 28 °C. Approx. 500 mg of cells were harvested in 2 ml collection tubes prior to substrate addition for 0 h readings. Glucosinolate (100 µM sinigrin) was then added and 100–500 mg cells were harvested at specific time points in 1.5 ml microcentrifuge tubes, washed thrice by adding 1 ml MSOT3, centrifugation at 4000 rpm, 30 s followed by discarding of supernatant and storage at − 80 °C. Kinetic experiments were performed directly in 2 ml safe lock microcentrifuge tubes. About 100 mg cells were added to 1 ml uptake buffer, mixed gently to create a suspension. The tubes were fixed on an orbital shaker at 130 rpm after substrates were added at specific concentrations, cells were harvested after 1 h and washed and stored as before.

### Glucosinolate extraction and HPLC analysis

Extraction of glucosinolates was done as mentioned in [11]. Briefly, the harvested cells were crushed in 70% methanol with sinalbin as internal standard (IS) in case of use of sinigrin and gluconapin as substrates, and sinigrin as IS where sinalbin was used as substrate was used for extraction, followed by incubation at 75 °C. The supernatant was loaded on 96 well plate containing Sephadex columns. The columns were washed with 70% methanol, twice with HPLC grade water and a final wash with 0.02 M MES buffer (pH 5.2). Finally, Sulphatase was added and plate was incubated at room temperature in dark for a minimum of 18 h. The glucosinolates in each well were eluted in HPLC grade water.

Samples were analyzed using Shimadzu CLASS—VP V6.14 HPLC machine. The program was set at a Solvent B (Acetonitrile) gradient of 1–19% through a 25 min cycle. The flow rate was maintained at 1 ml/min. and detection was made at 229 nm. The glucosinolates uptake by cells was determined by identifying the substrate peak by referencing it with known internal standard peaks.

### Intact glucosinolates extraction

Sinigrin hydrate was obtained from Merck, whereas gluconapin and sinalbin were extracted from *Brassica juncea* and *Sinapis alba* seeds, respectively using a previously described protocol [23]. Briefly, 20 gms of seeds was nicely crushed in 70% Methanol and centrifuged. Supernatant was transferred to fresh bottle and was re-extracted in 70% methanol. Supernatants from both extractions were combined and passed through Sephadex col. The column was washed three times with 2 ml formic acid/iso-propanol/water (3:2:5), four times with HPLC grade water. Glucosinolates were eluted into absolute ethanol through use of 0.5 M K_2_SO4/3% iso-propanol. Supernatant was evaporated; residue re-suspended in 70% methanol and kept at −20 °C for 30 min. The tube was centrifuged and supernatant was treated with three volumes of absolute ethanol. Pure glucosinolates was precipitated out through evaporation. The authenticity of the glucosinolate peaks has been described in our recent study [1] and a representative HPLC chromatogram has been provided as Additional file 1: Figure S1.

### Determination of kinetic parameters

One-way ANOVA, Tukey posthoc test was performed with SPSS software for comparison between the means of glucosinolate uptake at 100 µM of gluconapin, sinigrin and sinalbin, by vector controls, *AtGTR1* and *AtGTR2* expressing CCL1 lines. 3–4 clones showing comparable uptake were selected for each line for further kinetic analysis.

Kinetics graphs, average Km and Vmax values with standard errors were derived using Graphpad 6 using nonlinear regression analysis and fitting into the Michaelis–Menten equation. In order to study the effect of pH and nitrates in transport assay buffer, uptake data was subjected to student’s *t* test, two tailed distribution with a threshold *p* value of 0.05.

## Supplementary information

**Additional file 1: Figure S1.** HPLC chromatogram showing the peaks of purified glucosinolates used in this study. The identity of glucosinolates was based on similar retention time of peaks for sinigrin (SIN), sinalbin (4OHB) and gluconapin (GNA) using the method described in our recent study (Bajpai et al [1].

## Data Availability

All data generated or analyzed during this study are included in this published article.

## Abbreviations

CCL-1: Cotton cell suspension line-1
GTR: Glucosinolates transporter
HPLC: High performance liquid chromatography
MSOT3: MS media with additional KNO3 (1.9 g/L)
nmols/g/min: Nanomoles per gram per minute
qRT-PCR: Quantitative reverse transcription polymerase chain reaction
