# Supplementary material for: A cell suspension based uptake method to study high affinity glucosinolate transporters
Source: Plant Methods. 2020 May 24;16:75. doi: 10.1186/s13007-020-00618-0 (PMC7247208; doi:10.1186/s13007-020-00618-0)
Supplement: Supplementary file 1 — Additional file 1: Figure S1. HPLC chromatogram showing the peaks of purified glucosinolates used in this study. The identity of glucosinolates was based on similar retention time of peaks for sinigrin (SIN), sinalbin (4OHB) and gluconapin (GNA) using the method described in our recent study (Bajpai et al [1]. [file 13007_2020_618_MOESM1_ESM.pdf]

Supplementary data

Title: A cell suspension based uptake method to study high affinity glucosinolate transporters

Authors: Nambiar et al

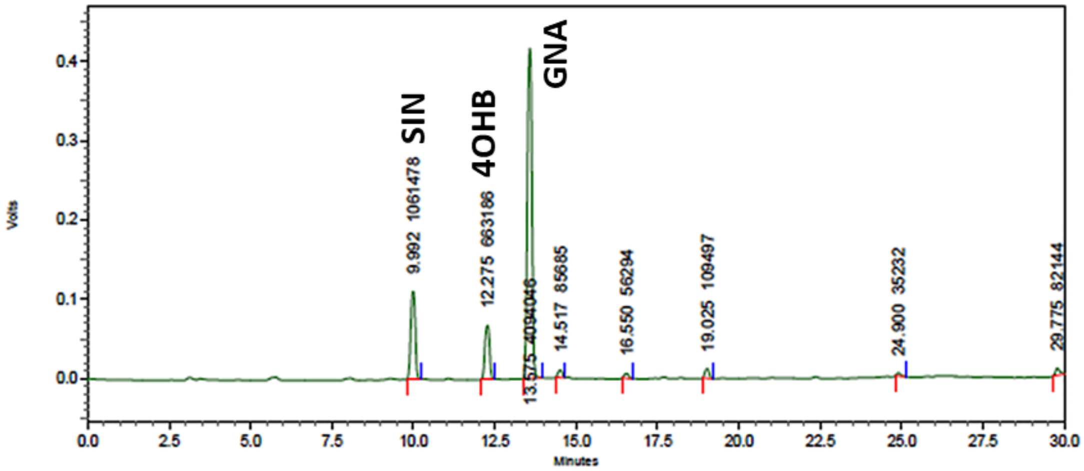

Figure S1: HPLC chromatogram showing the peaks of purified glucosinolates used in this study. The identity of glucosinolates was based on similar retention time of peaks for sinigrin (SIN), sinalbin (4OHB) and gluconapin (GNA) using the method described in our recent study (Bajpai et al., 2019).
